# 

**Published:** 1875-03

**Authors:** 


### CONTENTS:

ORIGINAL COMMUNICATIONS:

ART. I. Medical Expert and Medical Evidence—*continued.* By M. A. McClelland, M.D. .... 161

ART. II. Alcohol—its Theraputic Action and Remedial Agency. By Jas. S. Whitmire, M.D., Metamora, Ill. .... 172

ART. III. The best Treatment of Cancer. By J. E. Nichols, M.D., Osage, Iowa .... 179

PROGRESS IN MEDICAL SCIENCES:

Art. 1. Report on Practical Medicine. By N. Bridge, M.D., Chicago .... 189

ART. II. Progress of Obstetrics. By E. W. Sawyer, M.D., Chicago .... 194

ART. III. Progress of Surgery. By J. E. Owens, M.D., Chicago .... 197

REPORTS OF SOCIETIES:

Chicago Society of Physicians and Surgeons .... 202

SELECTIONS:

The Medical Year 1874 .... 205

EDITORS' BOOK TABLE .... 219

EDITORIAL .... 232

MORTALITY STATISTICS, Chicago .... 238

